# Peranal endoscopic myectomy for early rectal cancer with closure using a through-the-scope helical tack and suture system

**DOI:** 10.1055/a-2888-9922

**Published:** 2026-06-25

**Authors:** Tzong-Hsi Lee, Guan-De Li, Tai-Chuan Kuan, Keng-Li Lin, Chen-Shuan Chung

**Affiliations:** 1 Division of Gastroenterology and Hepatology, Departments of Internal Medicine 46608 Far Eastern Memorial HospitalNew Taipei CityTaiwan; 2 Division of Colon and Rectal Surgery, Department of Surgery 46608 Far Eastern Memorial HospitalNew Taipei CityTaiwan

A 70-year-old man underwent colonoscopy after a positive fecal immunochemical test, revealing a 3-cm rectal tumor located 4 cm above the anal verge ( Fig. 1a, b ). Abdominal computed tomography (CT) staged the lesion as cT2N0M0. Endoscopic ultrasound (EUS) suggested deep submucosal invasion with superficial involvement of the muscularis propria ( Fig. 2a, b ). Peranal endoscopic myectomy (PAEM) combined with focal endoscopic intermuscular dissection (EID) was performed to achieve en bloc resection. The mucosal defect was subsequently closed using a through-the-scope helical tack and suture system and endoclips ( Video 1 and Fig. 3a, b, c ).

**Fig. 1:**
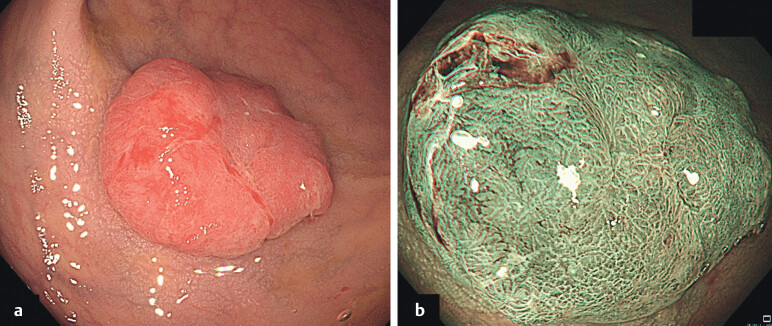
( **a** and **b** ) One 3 cm tumor (JNET type 2B with focal type 3) was found at the rectum, located 4 cm above the anal verge.

**Fig. 2:**
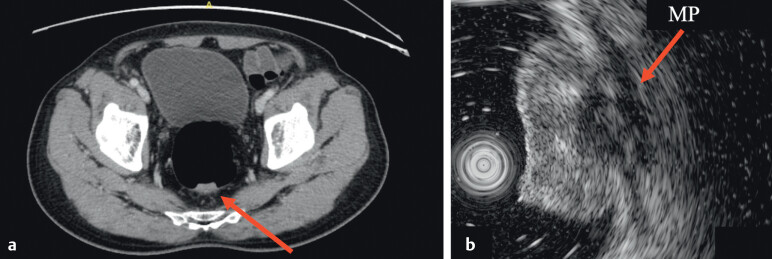
( **a** ) Abdominal CT demonstrated a rectal lesion with the clinical stage of cT2N0M0 according to the AJCC 8th edition. ( **b** ) EUS indicated tumor invasion into at least the deep SM layer with superficial involvement of the MP layer. MP, muscularis propria; SM, submucosal.

**Video 1** Peranal endoscopic myectomy (PAEM) for early rectal cancer with closure using a through-the-scope helical tack and suture system.


**Fig. 3:**
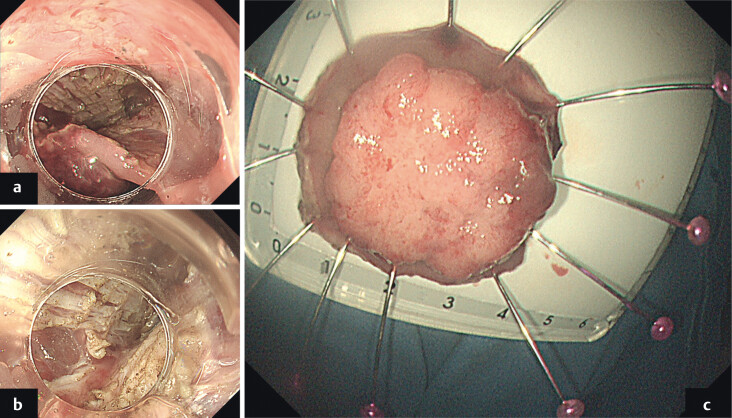
( **a** and **b** ) EID in between the inner (circular) part and the outer (longitudinal) part of the muscularis propria. ( **c** ) En bloc resection was achieved.

The postoperative course was uneventful, with no abdominal pain, hematochezia, or signs of peritonitis. Histopathology confirmed a moderately differentiated pT1 rectal adenocarcinoma with a submucosal invasion of >2 mm. Both horizontal and vertical margins were negative, with a 5 mm clearance ( Fig. 4a, b, c ). The patient subsequently received adjuvant concurrent chemoradiotherapy with oral capecitabine and radiotherapy (54 Gy in 30 fractions) in accordance with current guidelines. At 3-month follow-up, CT and colonoscopy demonstrated no evidence of local recurrence or distant metastasis.

**Fig. 4:**
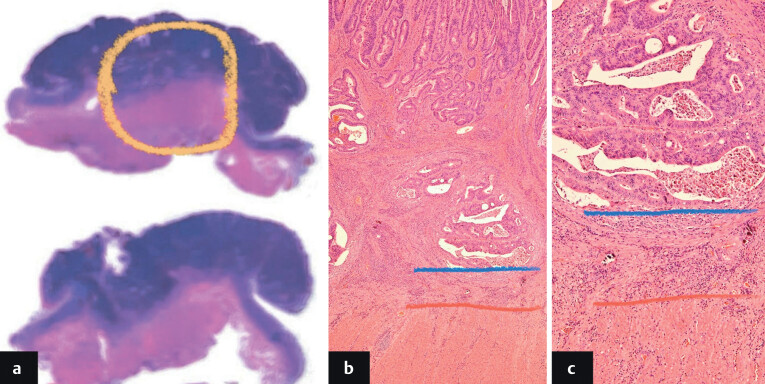
Final pathology confirmed rectal adenocarcinoma invading the submucosal layer (pT1), with both horizontal and vertical resection margins measuring 5 mm. ( **a** ) The upper purple part indicated mucosa, and the lower pink part indicated submucosa to muscularis propria. ( **b** and **c** ) The images represented low-power (b 4×) and medium-power (c 10×) magnification. The blue line indicated the lower border of the tumor, while the red line marked the upper margin of the muscular layer.

Endoscopic submucosal dissection achieves high R0 resection rates in superficial T1 cancers but is less effective for deep submucosal invasive cancer (D-SMIC). 1​ While total mesorectal excision remains the standard treatment, it carries significant morbidity. Emerging evidence suggests that PAEM may expand the role of endoscopic therapy to lesions previously considered unsuitable, including selected T2 tumors. 2​ EID enables dissection within the intermuscular plane, allowing deeper vertical margin clearance while preserving rectal wall integrity and surgical planes. Reported en bloc and R0 resection rates exceed 90% and 80%, respectively, in selected patients. 3​ ​4​

In this case, closure with the helical tack and suture system facilitated secure approximation of a large mucosal defect under tension, potentially reducing delayed adverse events such as bleeding or perforation. 5​ Notably, preoperative CT and EUS overstaged the tumor, likely due to inflammation or fibrosis, underscoring limitations in differentiating T1 from T2 disease.

PAEM combined with closure using a through-the-scope helical tack and suture system appears to be a feasible rectum-preserving strategy for selected patients with rectal D-SMIC, although careful selection and long-term outcomes remain essential.

Endoscopy_UCTN_Code_TTT_1AO_2AG_3AD

## References

[1] Watanabe D Toyonaga T Ooi M Clinical outcomes of deep invasive submucosal colorectal cancer after ESD Surg Endosc 2018 32 2123 2130 29098429 10.1007/s00464-017-5910-5

[2] Ichimasa K Kudo S E Hayashi T Potential indications for peranal endoscopic myectomy in lower rectal cancer Gastrointest Endosc 2025 101 1244 1247 39956466 10.1016/j.gie.2025.02.012

[3] Moons LM G Bastiaansen BA J Richir M C Endoscopic intermuscular dissection for deep submucosal invasive cancer in the rectum: a new endoscopic approach Endoscopy 2022 54 10 993 998 35073588 10.1055/a-1748-8573

[4] van der Schee L Albers S C Didden P Results of endoscopic intermuscular dissection for deep submucosal invasive rectal cancer: a three-year follow-up study Gut 2025 74 12 1995 2003 40562523 10.1136/gutjnl-2024-334612

[5] Mohapatra S Fukami N Follow-up outcomes of mucosal defect closures after endoscopic resection using a helix tacking system and endoclips VideoGIE 2022 7 268 272 35815167 10.1016/j.vgie.2022.03.002 PMC9263876

